# Whole exome sequencing identifies a novel missense *FBN2* mutation co-segregating in a four-generation Chinese family with congenital contractural arachnodactyly

**DOI:** 10.1186/s12881-016-0355-6

**Published:** 2016-12-03

**Authors:** Xingping Guo, Chunying Song, Yaping Shi, Hongxia Li, Weijing Meng, Qinzhao Yuan, Jinjie Xue, Jun Xie, Yunxia Liang, Yanan Yuan, Baofeng Yu, Huaixiu Wang, Yun Chen, Lixin Qi, Xinmin Li

**Affiliations:** 1 Shanxi Key Laboratory of Birth Defects and Cell Regeneration, Shanxi Population and Family Planning Research Institute, 11 Beiyuan Street, Taiyuan, Shanxi 030006 People’s Republic of China; 2 Department of Pathology and Laboratory Medicine, UCLA, Los Angeles, CA 90095 USA

**Keywords:** Whole exome sequencing, *FBN2*, Mutation, Congenital contractural arachnodactyly

## Abstract

**Background:**

Congenital contractural arachnodactyly (CCA) is an autosomal dominant rare genetic disease, estimated to be less than 1 in 10,000 worldwide. People with this condition often have permanently bent joints (contractures), like bent fingers and toes (camptodactyly).

**Case presentation:**

In this study, we investigated the genetic aetiology of CCA in a four-generation Chinese family. The blood samples were collected from 22 living members of the family in the Yangquan County, Shanxi Province, China. Of those, eight individuals across 3 generations have CCA. Whole exome sequencing (WES) identified a missense mutation involving a T-to-G transition at position 3229 (c.3229 T > G) in exon 25 of the *FBN2* gene, resulting in a Cys 1077 to Gly change (p.C1077G). This previously unreported mutation was found in all 8 affected individuals, but absent in 14 unaffected family members. SIFT/PolyPhen prediction and protein conservation analysis suggest that this novel mutation is pathogenic. Our study extended causative mutation spectrum of *FBN2* gene in CCA patients.

**Conclusions:**

This study has identified a novel missense mutation in *FBN2* gene (p.C1077G) resulting in CCA in a family of China.

## Background

CCA, also known as Beals-Hecht syndrome, is an autosomal dominant disorder of connective tissue. It was first described by Epstein et al. in 1968, and then was differentiated from Marfan syndrome (MFS) by Beals and Hecht in 1971 [1–3]. MFS and CCA share numerous common characteristics, such as a so-called marfanoid appearance constituted by tall, slender, asthenic appearance and skeletal features such as arachnodactyly, dolichostenomelia, pectus deformities, and kyphoscoliosis. In contrast with MFS, most individuals with CCA have “crumpled” ears, flexion contractures, and muscular hypoplasia.

Up to today, *FBN2* is the only gene known to be associated with CCA [4, 5]. The *FBN2* gene makes fibrillin-2 protein [6]. Fibrillin-2 is related to the elasticity of the tissue and plays a key role in the formation of extracellular microfibrils in elastic fiber providing strength and flexibility to connective tissue that supports the body’s joints and organs. Mutations in the *FBN2* gene can decrease fibrillin-2 production or result in the production of a fibrillin-2 protein with abnormal function. *FBN2* is expressed early in embryogenesis in elastic cartilage, aortic tunica media and bronchial epithelium [7]. *FBN2* deficient mice have forelimbs contractures, bilateral syndactyly and disorganized microfibrils [8].

As of September 5, 2016, 63 *FBN2* mutations have been described in 72 probands/families with CCA [6]. Because *FBN2*, located at 5q23.3, is a very large gene containing 65 exons and encoding 2912 amino acids, we thought many more mutations causative to CCA have yet to be identified. Here, we report a novel missense mutation in *FBN2* gene identified by whole exome sequencing in a four-generation CCA family.

## Case presentation

**Fig. 1:**
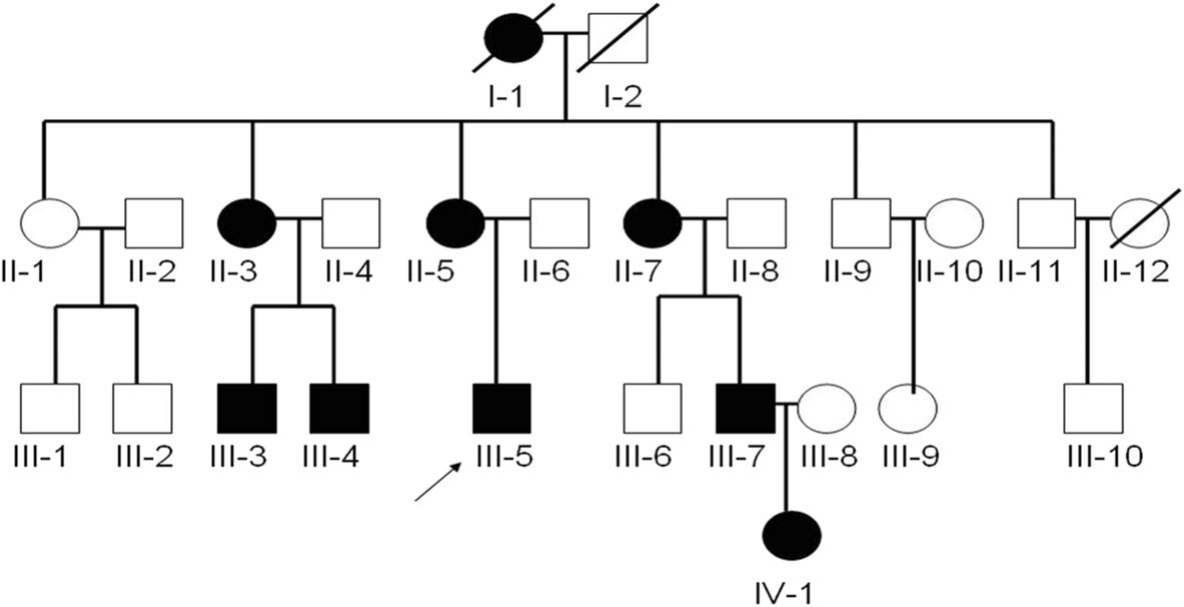
Pedigree of the family that took part in this study. The patients with congenital contractural arachnodactyly are depicted by black symbols. The arrow indicates the proband. The squares denote males and circles denote females. Crossed lines indicate deceased family members

The subjects of the study were 22 living members from a four-generation family affected with CCA living in the Yangquan County, Shanxi Province, China. The study was reviewed and approved by the institutional ethics committee of the Shanxi Population and Family Planning Research Institute in Shanxi, China. A written informed consent was obtained from the subjects before study. 7 ml peripheral venous blood was collected from 8 CCA patients (II-3, II-5, II-7 III-3, III-4, III-5. III-7, IV-1) and 15 normal subjects (II-1, II-2, II-4. II-6. II-8, II-9, II-10, II-11, II-12, III-1, III-2, III-6, III-8, III-9, III-10) in a Lavendar EDTA tube. The proband was the grandson III-5 (Fig. 1).

## Materials and methods

### DNA extraction

Genomic DNA was extracted from the blood samples using a DNA isolation kit for mammalian blood (Tiangen Biotech, China) according to manufacturer’s instructions. The integrity of DNA was evaluated using an Agilent 2100 Bioanalyzer (Agilent Technologies, Palo Alto, CA, USA) and purity/concentration was determined using a NanoDrop 8000 Spectrophotometer (Thermo Scientific, Wilmington, DE, USA).

### Sequencing and analysis

Whole exome sequencing was performed using Roche NimbleGen SeqCap EZ V3.0 System according to the manufacturer’s instruction. Briefly, 1ug of genomic DNA was sheared, size selected to roughly 300 bps. DNA library was made using Kapa LTP library preparation kit, followed by incubation with SeqCap biotinylated DNA baits. After an amplification of 14 cycles, the libraries were sequenced at 2X100 on a HiSeq 2500 sequencer.

The raw sequence data were aligned to the GRCh37 human reference genome using BWA v0.7.7-r411. PCR duplicates were marked using Mark Duplicates program in Picard-tools-1.115 tool set. GATK v3.2-2 and Samtools were used for the identification of INDEL and the SNVs, respectively. All variants were annotated using the Annovar program. FBAT (family based associated test) was used to identify SNPs that are significantly associated with CCA at the *p* value of 0.05.

## Results

### Clinical features of the patients

**Fig. 2:**
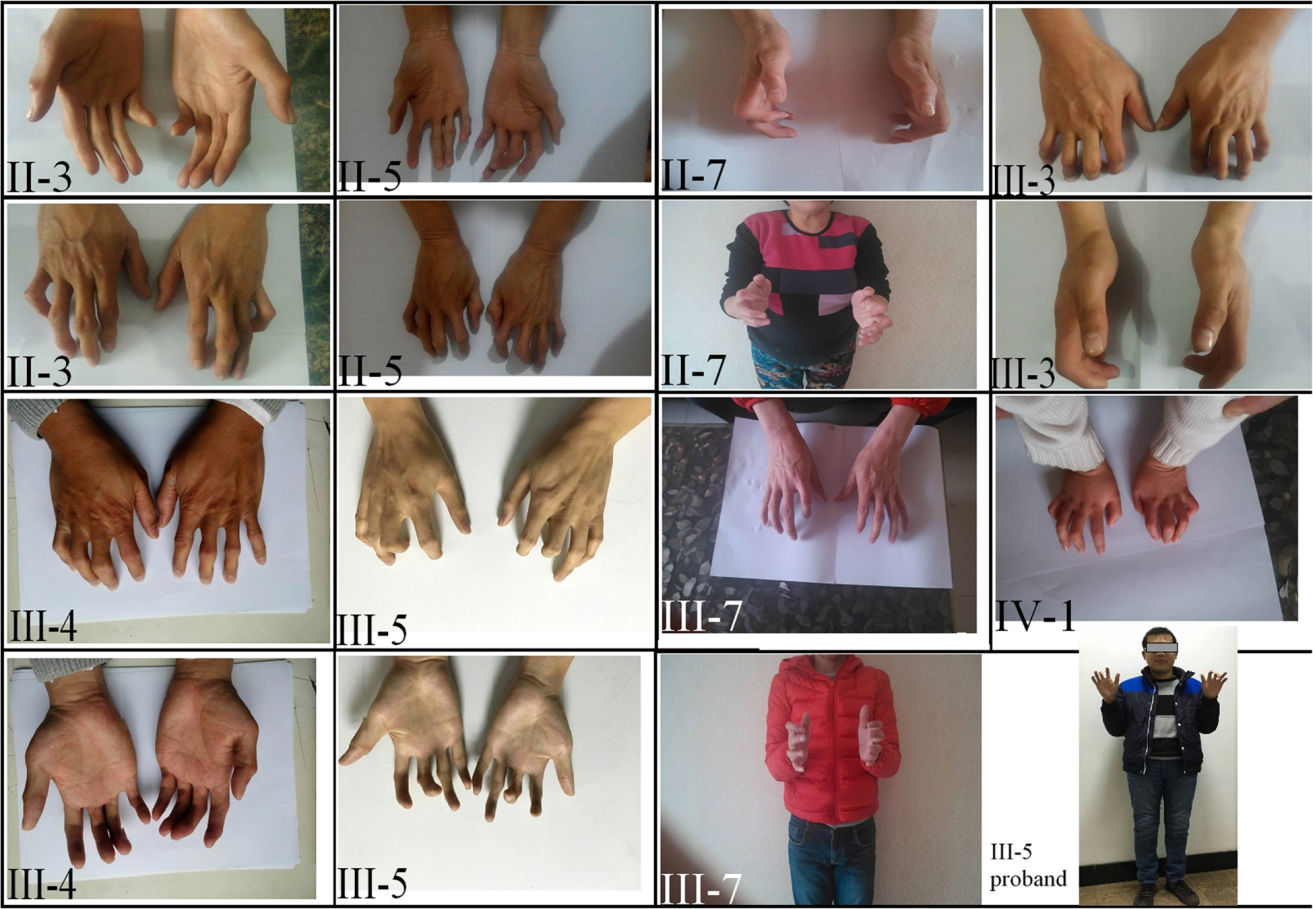
CCA phenotypes of eight affected family members. The photos of clubbed fingers from II-3, II-5, II-7, III-3; III-4, III-5, III-7 and IV-1 were taken when the samples were collected

The studied family has 25 members across four generations. Three of those were deceased (Fig. 1). A detailed physical examination was taken for 22 living members when samples were collected. Eight living members of the family have a typical CCA phenotype (II-3, II-5, II-7, III-3, III-4, III-5, III-7, IV-1) as presented with slender, contractural clubbed fingers and toes (Fig. 2). III-5 was the proband of 28 years old (at birth with clenched position of hands and 300 of myopia). No neurological, cardiovascular abnormalities, external ear malformation or eye abnormality were noted. Intrafamilial variation in phenotypic expression are modest.

### Mutation analysis

**Fig. 3:**
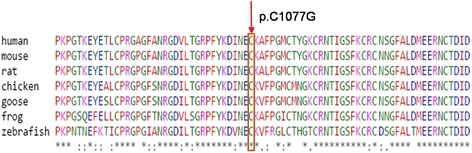
Protein sequencing alignment showing conservation of cysteine residue at the position 1077 in *FBN2* gene

**Fig. 4:**
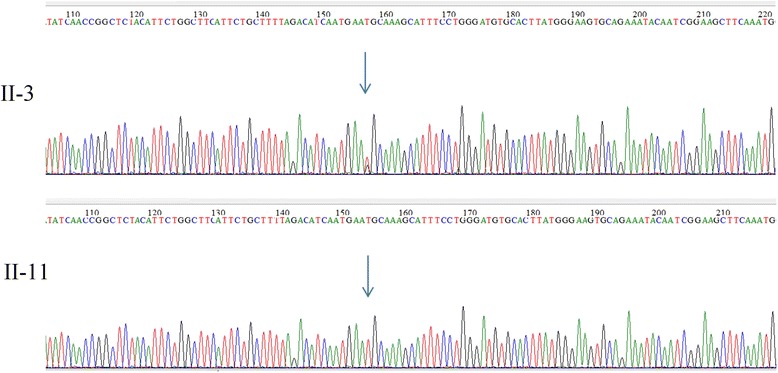
Sanger sequencing confirmation for the Mutation T3229Gin *FBN2* Gene

To reconcile clinical findings with molecular data in this family, whole exome sequencing was performed for all 22 living members with an Illumina Hiseq2500 sequencer. All samples were sequenced to a mean target coverage of >50x, and had a quality score of > Q30 for more than 85% of bases. After identification of all variant calls, FBAT analysis was used to identify variants that were significantly associated with CCA phenotype at p <0.05. Of those variants, heterozygous mutation, T3229G (chr5_127680191*,* p.C1077G) in *FBN2* gene, was the most significant one. This novel missense mutation was 100% co-segregated with the disease in the family and was absent in 14 unaffected members. C1077G mutation has a CADD PHRED score of 23.4. All six functional prediction tools (SIFT, PolyPhen-2, Mutation Taster, Mutation Assessor, FATHMM and FATHMM MKL Coding) predicted this mutation as damaging. Consistently, amino acid conservation analysis (http://genome.ucsc.edu) showed that the cysteine at position 1077 (p.C1077) is highly conserved across multiple species (Fig. 3). This mutation is also confirmed by Sanger sequencing (Fig. 4).

This variant was neither reported in global scale initiatives for variant annotation such as the Exome Aggregation Consortium (ExAC) nor described in the UMD-FBN2 database. However, one mutation located at the same amino acid position (p.Cys1077Arg) was documented in UMD-FBN2 database (reported as FRA01BOU F0061 I0092). Interestingly, the phenotype of this patient was also limited to joint contractures but with crumpled ears.

We also found a missense mutation in *FNIP1* gene, involving a A-to-G transition at position 2446 (p.I816V) in exon13, which was also 100% present in CAA patients and absent in all normal members in this family.

## Discussion

CCA is an established autosomal dominant genetic disease [9] characterized by contractures, arachnodactyly, dolichostenomelia, scoliosis, crumpled ears and pectus deformities [10]. The clinical symptoms are similar, but genetically distinct, to MFS. Mutations of *FBN2* gene cause CCAand mutations in the FBN1 gene result in MFS. However, MFS is genetically heterogeneous as it can be related to mutations in FBN1, TGFBR2 and TGFBR1 genes [11].

*FBN2* gene is located in chromosome 5q23-q31 and contains 65 exons, coding fibrillin-2 (2912 amino acids) [6]. For such a large gene, there are relatively small number of mutations that have been identified so far. As of May 20, 2016, ClinVar only recorded 336 mutations, of which 51 were considered as pathogenic by HGMD Professional database. Fifty-four percent of those pathogenic mutations are missense mutations, frequently replacing the Cysteine with another amino acid. The substitution of another amino acid for Cysteine can alter the structure or function of fibrillin-2 [12, 13]. All of these mutations reduce the amount of fibrillin-2 available to form microfibrils. Decreased microfibril formation will weaken the elasticity of fibers, which leads to the symptoms of CCA [14].

In this study, we found a novel missense mutation involving a T-to-G transition at position 3229 (c.3229 T > G) in exon 25 of *FBN2*, resulting in a Cys 1077 to Gly change (p.C1077G). Cys 1077 is highly conserved across many different species, suggesting it is required for the function of the protein. As expected, this mutation was predicted to be deleterious by SIFT and PolyPhne-2 software. Thus, this novel mutation is likely the genetic cause of CCA in this family.

Another missense mutation (rs7717874) in *FNIP1* gene, 100% co-segregated with CCA, has so far not been associated with any disease. It was predicted to be benign with a score of 0.002 (sensitivity: 0.99; specificity: 0.30) by PolyPhen-2. *FBN2* and *FNIP1* are only 310 kb apart, and thus the association between CCA and *FNIP1* mutation is likely due to the linkage disequilibrium. However, we cannot rule out a possibility that *FNIP1* gene has a regulatory role in the clinical expression of CCA.

## Conclusion

This study has identified a novel missense mutation in *FBN2* gene (p.C1077G) resulting in CCA in a family of China. The role of missense mutation at position 2446 (p.I816V) in *FNIP1* gene is worthy of further investigation.

## Abbreviations

CCA: Congenital contractural arachnodactyly
Cly: Clycin
Cys: Cysteine
DNA: Deoxyribonucleic acid
FBAT: Family‐based association test
FBN2: Fibrillin-2
FNIP1: Folliculin-interacting protein 1
HGMD: The human gene mutation Database
MFS: Marfan syndrome
PCR: Polymerase chain reaction
SIFT: Sorts intolerant from tolerant
SNP: Single nucleotide polymorphism
SNV: Single nucleotide variants
WES: Whole exome sequencing
